# Newly sequenced genomes of four Bacillus Calmette Guerin vaccines

**DOI:** 10.1590/0074-02760190401

**Published:** 2020-05-11

**Authors:** Maria Carolina Sisco, Marlei Gomés Silva, Beatriz Lopez, Claudia Arguelles, Leila Mendonça-Lima, Jacobus H de Waard, Rafael Silva Duarte, Philip Noel Suffys

**Affiliations:** 1 Universidade Federal do Rio de Janeiro, Instituto de Microbiologia Paulo de Góes, Departamento de Microbiologia Médica, Laboratório de Micobactérias, Rio de Janeiro, RJ, Brasil; 2 Instituto Nacional de Enfermedades Infecciosas, Buenos Aires, Argentina; 3 Instituto Nacional de Producción de Biológicos Carlos G Malbrán, Buenos Aires, Argentina; 4 Fundação Oswaldo Cruz-Fiocruz, Instituto Oswaldo Cruz, Laboratório de Genômica Funcional e Bioinformática, Rio de Janeiro, RJ, Brasil; 5 Servicio Autónomo Instituto de Biomedicina Dr Jacinto Convit, Caracas, Venezuela; 6 One Health Research Group, Universidad de Las Américas, Facultad de Ciencias de la Salud, Quito, Ecuador; 7 Fundação Oswaldo Cruz-Fiocruz, Instituto Oswaldo Cruz, Laboratório de Biologia Molecular Aplicada às Micobactérias, Rio de Janeiro, RJ, Brasil

**Keywords:** mycobacteria, BCG, whole genome sequencing.

## Abstract

Bacillus Calmette Guerin (BCG) vaccines comprise a family of related strains. Whole genome sequencing has allowed the better characterisation of the differences between many of the BCG vaccines. As sequencing technologies improve, updating of publicly available sequence data becomes common practice. We hereby announce the draft genome of four commonly used BCG vaccines in Brazil, Argentina and Venezuela.

*Mycobacterium bovis* Bacillus Calmette Guerin, commonly known as BCG, is the only vaccine against tuberculosis. The original BCG strain was obtained by serial passages of a *M. bovis* strain in potato-bile media.^1^ Deletion of the region of difference (RD) 1 was later confirmed as one of the reasons for the attenuation of its virulence.^2^ ^,^ ^3^ After its first use in humans, the vaccine was sent to different laboratories worldwide where different culturing conditions originated strains with different genetic compositions.^4^

At present, there are more than 10 different vaccine strains being administered worldwide.^5^ In two countries in Latin-America, namely Venezuela and Argentina, the strains BCG Danish 1331 (Statens serum Institut, Denmark), BCG Pasteur 1173P2 (Instituto Nacional de Producción de Biológicos ― ANLIS Carlos G Malbrán, Argentina) and BCG Sofia SL222 (BB NCIPD Ltd, Bulgaria) are licensed for use. The vaccine BCG Pasteur produced in Argentina is a secondary seed lot of the French BCG Pasteur strain 1173P2 and is administered in the Province of Buenos Aires, while the rest of the country is vaccinated either with the Sofia or the Danish strain. In Brazil, BCG Moreau RDJ (Fundação Ataulpho de Paiva, Brazil) was used as a vaccine until 2017, when it was replaced by the Russian strain.

Whole genome sequencing data of the strains Moreau, Pasteur and Danish are already available^6^ ^,^ ^7^ ^,^ ^8^ ^)^ and obtained either by using shotgun sequencing and specific primers designed to close the gaps in the assembly (for Moreau and Pasteur strains) or a combination of Illumina and PacBio technology (for the Danish strain). BCG Sofia has so far only been subjected to whole genome analysis using microarrays.^9^ We sequenced the genome of these four vaccine strains with Illumina technology in an effort to update the sequencing data available and for BCG Sofia, we report the first sequence data obtained with newer technology.

Genome sequencing of the four vaccine strains was performed using the Nextera XT DNA Library preparation kit on an Illumina HiSeq 2500 platform. *De novo* assembly was done using Unicycler^10^ and annotated with RAST.^11^ To determine intra-strain genomic variability of each vaccine, we compared the genomes with previous assemblies obtained from the NCBI^6^ ^,^ ^7^ ^,^ ^8^ using the software Artemis Comparison tool^12^ and Snippy.^13^ The strain BCG Sofia SL222 originated from the Russian vaccine BCG-1 and was chosen as a master seed at the BCG Bulgarian laboratory.^9^ Because there is no whole genome assembly available for BCG Sofia SL222, we decided to use the assembly of its parental strain BCG-1 Russia for the comparative studies.^14^

Among the four genomes, we obtained between 82 and 108 contigs, an average guanine-cytosine content (GC) of 65%, a size ranging between 4.2 and 4.3 Mb and the number of coding sequences (CDS) between 4205 and 4245 (Table I). The differences in the size of BCG strains genomes we noticed when compared to those available in public databases is probably due to variation in sequencing technologies and of assemblers used.

**TABLE I:** Assembly statistics for the four vaccine strains sequenced

Number of contigs	Moreau RDJ	Pasteur 1173P2	Sofia SL222	Danish 1331
	82	93	102	108
Genome size (bp)	4288.245	4.192.545	4.201.889	4.202.807
Coverage	414X	107X	101X	94X
% GC	65.62	65.48	65.45	65.47
N50	197411	84414	70691	70718
CDS	4232	4205	4245	4227
tRNAs	47	47	47	47

bp: base-pairs; %GC: guanine-cytosine content; CDS: coding sequences; tRNA: transfer RNA.

The genome of BCG Moreau RDJ strain revealed 55 single nucleotide polymorphisms (SNPs) compared to that of the shotgun sequencing based genome of the same strain obtained in 2011, 28 of these SNPs are non-synonymous (ns) (Table II). We also detected five insertions and four deletions of 3-4 nucleotides (data not shown) and an inverted IS1608 transposase gene (position 3717335-3717826 bp).

**TABLE II:** Non-synonymous single nucleotide polymorphisms (SNPs) found in Bacillus Calmette Guerin (BCG) Moreau when using assembly NZ_AM412059^*^ as a reference

Position	NZ_AM412059	BCG Moreau	AA change	Gene
404956	T	G	Glu713Asp	Iron-sulphur-binding reductase
555536	C	T	Gly164Glu	FIG00821074: hypothetical protein
555569	G	A	Pro153Leu	FIG00821074: hypothetical protein
570675	T	G	Lys91Asn	Aliphatic amidase AmiE
878380	G	T	Gly233Val	Protease II (EC 3.4.21.83)
1217552	T	G	His65Gln	PE family protein
1618404	A	G	Asp284Gly	Anaerobic dimethyl sulfoxide reductase chain A
1618472	A	C	Met307Leu	Anaerobic dimethyl sulfoxide reductase chain A
1618722	G	C	Arg390Pro	Anaerobic dimethyl sulfoxide reductase chain A
1618779	T	G	Val409Gly	Anaerobic dimethyl sulfoxide reductase chain A
1731072	G	A	Ala234Thr	Sorbitol-6-phosphate 2-dehydrogenase
1985896	C	G	Pro114Ala	L-gulono-1,4-lactone oxidase
2400116	A	G	Leu184Pro	Cell division protein FtsL / proline rich membrane protein
2651260	C	G	Ala266Gly	PE family protein
2701298	G	T	Pro413Thr	Ribonuclease E
2760281	T	C	Ser266Gly	GTP-binding protein Obg
2760610	G	C	Ala156Gly	GTP-binding protein Obg
2760682	T	C	Glu132Gly	GTP-binding protein Obg
3149570	C	G	Leu224Val	Coenzyme F420-dependent oxidoreductase
3273878	A	G	Val602Ala	ATP-dependent DNA helicase RecG
3365033	A	C	Trp93Gly	Transcriptional regulator, TetR family
3809510	C	G	Gly67Ala	FIG00820542: hypothetical protein
3879667	A	G	Asn344Asp	GTP-binding protein Obg
3881120	T	G	Ile828Ser	GTP-binding protein Obg
3881141	C	A	Thr835Asn	GTP-binding protein Obg
3891798	T	G	Asp162Ala	Long-chain fatty-acid-CoA ligase Mycobacterial subgroup FadD19
3963021	C	G	Val222Leu	Transcriptional regulator, LacI family
4172275	T	G	Met67Leu	Membrane proteins related to metalloendopeptidases

*: accession number for the assembly of BCG Moreau reported by Gomes et al.^(6)^ A: adenine; G: guanine; C: cytosine; T: thymine; Glu: glutamic acid; Asp: aspartic acid; Gly: glycine; Pro: proline; Leu: leucine; Lys: lysine; Asn: asparagine; Val: valine; His: histidine; Gln: glutamine; Met: methionine; Arg: arginine; Thr: threonine; Ser: serine; Ala: alanine; Trp: tryptophan; Ile: isoleucine.

Upon sequencing BCG Sofia SL222 and after comparison with the BCG-1 Russian strain, we observed one synonymous (s) SNP in the gene coding for an uridylyltransferase, in addition to three inverted regions of 42,965 bp, 17,778 bp and 6,765 bp in length. Furthermore, by mapping the reads obtained from the Sofia strain to the genome of the Danish vaccine strain, we confirmed the presence of the 1.6 kb deletion described by Stefanova et al.^9^ This deletion affects part of the gene coding for type II toxin-antitoxin system VapC family toxin, the gene for the antitoxin VapB48 and part of the glutamate - cysteine ligase gene.

The genome of BCG Danish 1331 was the last to be assembled by using a combination of Illumina and PacBio reads.^7^ One advantage of performing PacBio sequencing is that it generates longer reads that improves detection of repeated regions and duplications. Upon sequencing, we observed five SNPs including four nsSNP and a stop codon (Table III). We also observed a deletion of five nucleotides in a SRPBCC family protein gene and two inversions of 26,170 bp and 7,565 bp.

**TABLE III:** Non-synonymous single nucleotide polymorphisms (SNPs) found in Bacillus Calmette Guerin (BCG) Danish when using assembly NZ_CP039850^*^ as a reference

Position	NZ_CP039850	BCG Danish	AA change	Gene
593769	C	T	Gln323**	SDR family oxidoreductase
2076695	C	A	Ala142Ser	M56 family metallopeptidase
2500583	T	C	His260Arg	Sulfotransferase
3745609	G	T	Ser434Tyr	PPE family protein
3839864	T	G	Thr135Pro	IMP dehydrogenase

*: accession number for the sequencing of BCG Danish reported by Borgers et al.^(7)^; **: indicates a stop codon; A: adenine; G: guanine; C: cytosine; T: thymine; Gln: glutamine; Ala: alanine; Ser: serine; Thr: threonine; Pro: proline; Tyr: tyrosine.

Genome assembly of BCG Pasteur presented a nsSNP in the GTP-binding protein Obg gen (Asn599Asp) and two inframe insertions of three nucleotides each in the genes coding for NADPH epimerase/NADPH dehydratase and a probable cutinase. We also found one inverted region of 31,516 pb.

*De novo* sequencing of genomes deposited in public databases becomes imperative as new sequencing technologies arise. Recently, Abdallah et al.^15^ reviewed the genomes and transcriptomes of fourteen BCG vaccine strains and together with the work of Borgers on the Danish vaccine comprise the most recent studies in BCG strains genealogy. We announce the initial draft genome of four of the most common BCG vaccines licensed worldwide in an effort to contribute to the update of publicly available data. The comparative analysis of BCG strains remains of crucial importance to trace their divergence in terms of genetic sequence, transcription and proteomic profile and, subsequently, to describe possible variation in the protective efficacy.

*Accession numbers* - The reads of each genome have been deposited under SRA accession PRJNA575846, BioProject ID: PRJNA575846.
